# Integrating multiple technologies to understand the foraging behaviour of Hawaiian monk seals

**DOI:** 10.1098/rsos.160703

**Published:** 2017-03-08

**Authors:** Kenady Wilson, Charles Littnan, Patrick Halpin, Andrew Read

**Affiliations:** 1 Duke University Marine Lab, 135 Duke Marine Lab Rd, Beaufort, NC 28516, USA; 2 Pacific Island Fisheries Science Center, 1845 WASP Blvd., Building 176, Honolulu, HI 96818, USA; 3 Nicholas School of the Environment, Duke University, 9 Circuit Drive, Durham, NC 27708, USA

**Keywords:** Hawaiian monk seal, foraging behaviour, accelerometer, Crittercam

## Abstract

The objective of this research was to investigate and describe the foraging behaviour of monk seals in the main Hawaiian Islands. Specifically, our goal was to identify a metric to classify foraging behaviour from telemetry instruments. We deployed accelerometers, seal-mounted cameras and GPS tags on six monk seals during 2012–2014 on the islands of Molokai, Kauai and Oahu. We used pitch, calculated from the accelerometer, to identify search events and thus classify foraging dives. A search event and consequent ‘foraging dive’ occurred when the pitch was greater than or equal to 70° at a depth less than or equal to −3 m. By integrating data from the accelerometers with video and GPS, we were able to ground-truth this classification method and identify environmental variables associated with each foraging dive. We used Bayesian logistic regression to identify the variables that influenced search events. Dive depth, body motion (mean overall dynamic body acceleration during the dive) and proximity to the sea floor were the best predictors of search events for these seals. Search events typically occurred on long, deep dives, with more time spent at the bottom (more than 50% bottom time). We can now identify where monk seals are foraging in the main Hawaiian Islands (MHI) and what covariates influence foraging behaviour in this region. This increased understanding will inform management strategies and supplement outreach and recovery efforts.

## 1. Introduction

Hawaiian monk seals (*Neomonachus schauinslandi*) are one of the most endangered seal species in the world. They are the last surviving species in their genus with abundance declining by roughly 3.4% per year [1]. The majority of the species (approx. 900) resides in the remote and protected Northwestern Hawaiian Islands (NWHI) with a small subpopulation (approx. 200) in the main Hawaiian Islands (MHI). The MHI population is doing well with abundance increasing by 6.5% annually [1,2] and current trends suggest that abundance in the MHI will equal that in the NWHI within the next 10 years [1]. One hypothesis for this increased survival is that it is easier for seals in the MHI to find and capture prey than their counterparts in the NWHI. Understanding the variables that affect foraging behaviour is often crucial for the successful recovery of endangered species. However, before we can examine the ecological drivers of foraging behaviour, we first need to identify when and where foraging events occur.

It is difficult to observe foraging events directly in aquatic predators, so researchers often rely on inference from biologging instruments. For several decades, diving vertebrates have been equipped with instruments that record time and depth, allowing the reconstruction of two-dimensional dive profiles. Classification of these two-dimensional dive shapes has been used to categorize diving behaviours in many marine vertebrates, leading to inferences regarding the function of different dive types (i.e. foraging versus travelling) [3–6]. In this type of analysis, dive profiles are generally characterized as one of four shapes: square, V, skewed right and skewed left [7], with behaviour inferred from these shapes. Some researchers have used direct evidence of feeding, such as stomach temperature telemetry [8,9], jaw movement [10,11] and video [12,13] to infer functions of different dive shapes or bout types. For example, many studies have demonstrated that square dives (vertical travel to the bottom, travel along the sea floor and a vertical return to the surface) commonly occur during foraging. Nevertheless, uncertainty remains in the interpretation of two-dimensional dive behaviour, which makes it difficult to identify searching and consumption in multiple dives.

Marine mammals live in a three-dimensional environment, so studies of their foraging behaviour should incorporate movement in all three dimensions. Dive classification based on three-dimensional movements is a relatively new technique that can provide a more accurate description of fine-scale movements of diving animals. For example, acoustic tracking of the three-dimensional movements of Weddell seals (*Leptonychotes weddellii*) demonstrated that classification of dive types based on time and depth alone oversimplified a complex suite of behaviours [14]. Acoustic tracking describes movements in greater detail than two-dimensional dive classification, but does not identify specific behavioural states or provide inference on the ecological functions of different dive types [14–16]. Three-axis accelerometers measure animal orientation and the dynamics of movement [17,18], enabling the reconstruction of three-dimensional diving behaviour in marine animals [18–20]. A complete suite of parameters, including compass bearing, time, depth, accelerometry and video recording, would allow for the reconstruction of three-dimensional dives and identify the function(s) of each dive [13,21–23]. This combination is, thus far, the only approach that simultaneously records and validates the function of the behaviour of a diving animal.

The first comprehensive study of Hawaiian monk seal foraging ecology in the MHI was completed by Cahoon [24] who tagged 18 seals with satellite-linked dive recorders. These seals foraged primarily in depths less than 200 m near the islands. In a follow-up study, Wilson [25] deployed GPS-GSM phone tags (Sea Mammal Research Unit, St Andrews, Scotland) in the MHI and demonstrated that seals spend most of each day at sea, with less than 40% of each day hauled out on land. These initial results showed that seals in the MHI had shorter foraging trips than their counterparts in the NWHI (mean trip duration less than 1 day versus 17 days) and provided a general understanding of monk seal movements in the MHI, but did not allow for a fine-scale interpretation of at-sea behaviour. Wilson [25] also showed that monk seals travelled continuously along the sea floor during foraging trips, exhibiting one main dive type (square-shaped and benthic). This dive type is often used to infer foraging, which suggests that monk seals may be actively searching for food the entire time they are at sea. However, without a way to identify dives that contained prey-capture attempts, or active search events, it was impossible to test this hypothesis directly.

Recent advances in recording technology and miniaturization of instruments have allowed scientists to attach multiple instruments to a single animal without dramatically affecting the dive behaviour [11,26]. These instruments document the fine-scale behaviour of diving animals through inertial motion sensors, the behaviour of both predator and prey through video and, in some cases, measurements of the physical environment such as temperature [13,27,28]. The overall goal of our study was to define a reliable metric of foraging for monk seals (including both searching for and consuming prey) and to examine the environmental and behavioural variables that may influence foraging. To do this we used a combination of GPS telemetry (GSM phone tag), animal-borne video (National Geographic Crittercam) and three-dimensional inertial motion sensors (Open Tag) to record the underwater behaviour of Hawaiian monk seals. Similar to Watanabe and Takahashi [22], we first observed foraging behaviour in the video footage, looking at what, where and how monk seals captured prey. Second, we used a signal from the accelerometer to identify foraging behaviours and validated the signal using the video footage. Lastly, we extended the signal analysis to each seal's full data record to examine the environmental and behavioural influences on foraging events in the region.

## 2. Methods

### 2.1. Instrument deployment

**Figure 1.**
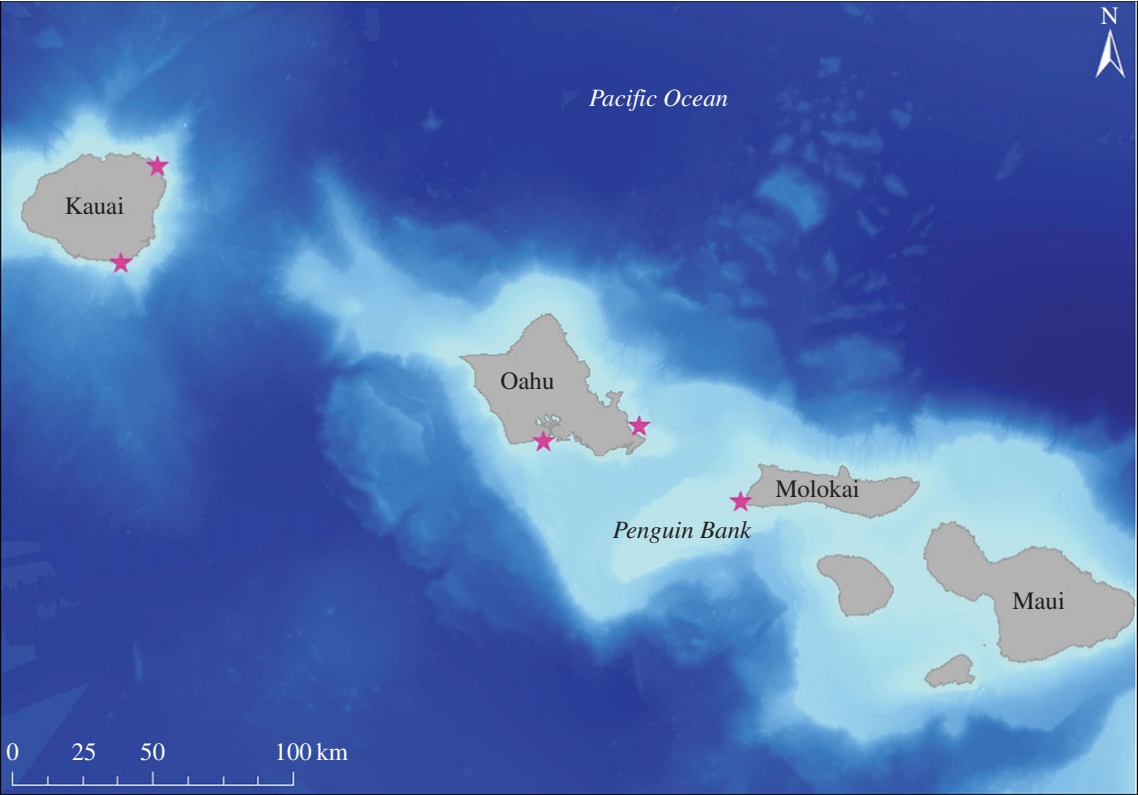
Capture locations of Hawaiian Monk Seals in the main Hawaiian Islands. Three seals were instrumented on Oahu, nine on Molokai and four on Kauai.

**Figure 2.**
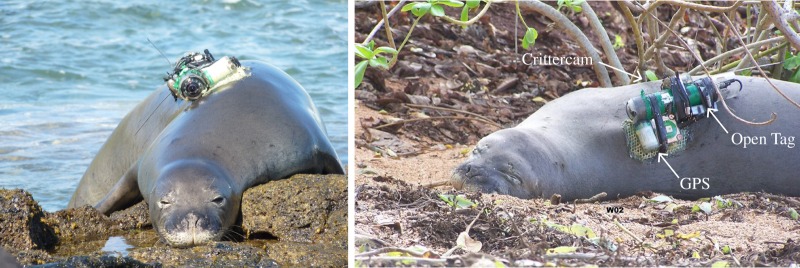
Photo of the instrument package attached to a Hawaiian monk seal. The package includes a National Geographic Crittercam, a Loggerhead Instruments Open Tag and a SMRU GPS phone tag.

We captured sub-adult and adult seals on Oahu (*n* = 3), Molokai (*n* = 9) and Kauai (*n* = 4) (figure 1) following the methods of Baker & Johanos [29]. Due to capture guidelines that restrict the capture of pregnant or potentially pregnant females [29], we only instrumented one female during the first year of fieldwork. For the remainder of the study, therefore, we targeted sub-adult and adult males. Seals were captured on the beach with a hoop net and sedated with Diazepam (5 mg ml^−1^ at 0.1–0.25 mg kg^−1^ IV). The instrument package included a National Geographic Crittercam, Loggerhead Instruments Open Tag (3-axis accelerometer, magnetometer, gyroscope and pressure sensor), a Sea Mammal Research Unit GSM phone tag and a VHF transmitter (table 1). We glued the package to the pelage of the animal, along the dorsal midline between the shoulder blades using 10 min epoxy (Devcon, Danvers, MA, USA) (figure 2). GPS tags were programmed to record a position estimate every 20 min unless the seal was underwater or hauled out on land [25]; the Open Tags recorded continuously on all sensors at 100 Hz until the battery was exhausted (approximately 4–6 days). We programmed Crittercams to record for 30 min of every 2 h cycle between the hours of 8.00 and 17.00 (Hawaii Standard Time), when the camera was wet. Four to 6 days after deployment, we used the GPS and VHF tags to find and recapture the seals to recover the Crittercam and Open Tag; the GPS tag was left on the animal to collect long-term movement and summary dive data for an additional 3–6 months.

**Table 1.** Description of tags deployed on Hawaiian monk seals as part of the instrument package.

instrument	company	data	data recovery method	deployment duration
GSM-GPS phone tag	Sea Mammal Research Unit	dive depth, duration, and location. Haul-out times and locations	transmitted though the GSM cell phone network	3–6 months
Crittercam	National Geographic	video of prey and time of prey capture attempts and successes	must be recovered	3–6 days
Open Tag	Loggerhead Instruments	gyroscope, magnetometer, acceleration, and depth	must be recovered	3–6 days

### 2.2. Data processing

We processed the GPS data using methods described in Wilson [25]. Briefly, GPS data were visually inspected and erroneous locations were removed. Only GPS data collected while both the Open Tag and Crittercam were recording are included here. We matched Open Tag, GPS and Crittercam records using dive start times, surface events (time spent at the surface) and dive depth recorded by all three instruments. We accounted for any drift by comparing the date and time of the deepest dive in each trip. We identified the time of the deepest dive of the trip on each instrument and then used the clock offset between the instruments to readjust the time so that everything matched the GPS data record.

The Open Tag data were analysed in Python to reconstruct the full dive record and describe the following behavioural variables for each dive: dive depth and duration; pitch, yaw and roll; root mean square (RMS) of rotational velocity; mean overall dynamic body acceleration (ODBA); the presence of active search events; proportion of dive spent searching; and bottom time. Bottom time was defined as the amount of time spent at more than 80% of the maximum depth of the dive. We used a moving average of 2 s to filter out the gravity signal in each of the accelerometer axes before calculating ODBA. We then followed the methods of Wilson *et al*. [30] for the calculation: ODBA=abs(X)+abs(Y)+abs(Z). Roll and pitch were calculated from the raw accelerometer recordings and yaw was calculated from the magnetometer after removing the roll and pitch from the magnetometer readings. For the axes, *X* is along the body (across the shoulders of the seal), *Y* is normal to the *X* in the horizontal plane (nose to tail) and *Z* is normal to the horizontal plane, through the long body axis (dorsal to ventral). Specifically, roll is equal to the arctan2 of the *Y* and *Z* accelerometer readings. After roll is removed from the accelerometer recording, pitch is calculated as the arctan of the negative *X* acceleration divided by de-rotated *Z* acceleration. Yaw is calculated as the arctan2 of the magnetometer *Y* and *X* values after removing the roll and pitch from the magnetometer values. The gyroscope measures rotational velocity and hence the signal from those data is directly related to fluking in Phocids. The RMS of the rotational velocity is a good way to measure the average energy in the gyroscope signal because it oscillates around 0, and was therefore used as a proxy for average stroking movement per dive, and an additional measure of body motion during the dive.

We assigned environmental variables, including bathymetry, distance to shore, bottom complexity and habitat type to each dive based on GIS analysis. Using bathymetry and dive depth, we also calculated the ratio of dive depth to the bathymetry where the dive occurred (dive ratio). Environmental variables were extracted from the centre of a 1 km grid, overlaid on the study area. Bottom complexity was composed of three individual variables produced using the Benthic Terrain Modeler program in ArcGIS: slope, terrain ruggedness and rugosity [21]. Habitat type was assigned by extracting the benthic habitat (pavement, sand, mud, coral, rubble etc.) at the location of the start of each dive. Habitat data were obtained from NOAA's Center for Coastal Monitoring and Assessment (CCMA) [31], which mapped shallow-water (less than 30 m) habitats around the MHI. According to the CCMA database, all of the dives that occurred on Penguin Bank, a monk seal foraging area near Molokai (figure 1), occurred in ‘Unknown’ habitat. This was because Penguin Bank is more than 30 m in depth and the habitat assessment was for waters less than 30 m. There were no habitat assessments available for deep-water regions. Consequently, we manually defined the ‘habitat’ for dives occurring on the Bank as ‘Penguin Bank’. This was done to separate truly unknown shallow-water habitat from Penguin Bank habitat.

### 2.3. Identifying search events

Monk seals are benthic foragers and typically target cryptic prey that hides in the sand or under rocks [25,32–34]. Video footage revealed that monk seals invert their bodies in a head-down position when searching for prey. While inverted, they either investigate an area quickly and move on, or investigate more thoroughly by digging in the sand, blowing bubbles or flipping/shoving rocks with their head. Some prey captures were visible in the video footage (*n* = 10), particularly octopus and large fish, but capture success when targeting smaller prey was not confirmed (*n* = 13) due to sand or bubbles billowing around the camera, or the prey being too small to be seen while the seal's head was under a rock. For this reason, and since this is the first study of its kind for this species, we decided not to distinguish between search events and prey-capture attempts. Instead, we focused on broader foraging patterns and defining a method that would reveal when and where all foraging/search events occurred.

Every prey capture or attempted capture we observed on the video occurred from an inverted body position. Therefore, pitch, which is related to movement along the head–tail body axis, was an ideal metric to identify this head-down position and consequent search or feeding event. However, monk seals also have a steep descent at the start of each dive and swim in a near-vertical position for the first few metres. To eliminate the possibility of identifying the start of a dive as a search event, we added depth to the pitch metric and identified a search event whenever the pitch of the animal was greater than or equal to 70° and depth was less than or equal to −3 m. Using the pitch metric, we also calculated the amount of time spent searching on each dive (i.e. the amount of time seals spent at or above a pitch angle of 70° while diving below 3 m). This was done to look at the spatial representation of foraging and to see if more effort was focused in particular regions.

The 70° threshold was initially chosen at random after visually observing the data. It was verified by animating the pitch axis of the accelerometer and viewing the animation with synoptic video footage (electronic supplementary material, S1). We then matched 93 dives to concurrent video footage. If at least one search event occurred on a dive, that dive was classified as a searching/foraging dive. Search events were identified from the video footage (observed) or a peak in the pitch axis of the accelerometer (predicted). We validated the pitch metric by comparing the observed (video) and predicted (pitch) dive classification for all dives that were matched to concurrent video footage. By comparing the predicted classification to the observed video data, we could identify false-positive and false-negative prediction for the pitch metric.

### 2.4. Model selection

We used behavioural and environmental variables to predict the occurrence of search events for monk seals. We designated a *searching dive* as one containing at least one search event (with pitch greater than or equal to 70° and dive depth less than or equal to −3 m). Model selection was done using a Bayesian generalized linear mixed model with a binomial likelihood [35]. There was a considerable degree of individual variation in behaviour (K. Wilson 2015, unpublished data) [25], so we modelled each seal separately before adopting a mixed model.

The response variable was dive type (searching or not). The fixed effects in the global models were: maximum dive depth (m); dive duration (s); RMS of rotational velocity (RMS gyro); mean ODBA; proportion of time spent at the bottom of the dive (bottom%); bathymetry (m); the ratio of dive depth to bathymetry (dive ratio); distance to shore (m); slope; terrain ruggedness (variation in three-dimensional orientation of the grid cells within a neighbourhood); rugosity (ratio of surface area to planar area); and habitat where the dive occurred (pavement, sand, aggregate reef, rock, boulder, pavement with channels etc.). We selected variables by comparing AIC, BIC, null and residual deviance values and chose the most parsimonious model (excluding functionally redundant or highly correlated variables) with the lowest BIC values as the best fit. The variables selected for all individual seals included some measure of depth (dive or bathymetry), dive duration and movement (ODBA or RMS gyro). This suggested that a mixed model could account for the variation among individual seals. We then compiled the data and model selection was performed again, starting with the variables in the global model, and seal as a random effect. After variable selection for the mixed model was complete, we used a Gibbs sampler to model the data using a binomial likelihood and uninformative priors on the predictors. The model for dive *j* = 1, … , *n* was

2.1 $$y_{ij}∼\text{Bernoulli}(\theta_{ij}).$$

With an inverse link function to map the parameters,

2.2 $$\text{logit}(\theta_{ij})=x_{ij}\beta +\alpha_{k}.$$

Here *Y_ij_* = 1 is the event that a search was identified on a dive, *θ_ij_* is the probability associated with a search event on dive *ij*, *x_ij_* is the design matrix of predictor variables, *β* are the coefficients associated with each fixed predictor and *α_k_* is a random intercept for seal *k*. The prior distributions for *β* and *α* were normal:

2.3 $$\begin{array}{rl} \beta & ∼\text{Normal}(\mu_{\beta },\varphi_{\beta }), \end{array}$$

2.4 $$\begin{array}{rl} \alpha_{k} & ∼\text{Normal}(\mu_{\alpha },\varphi_{\alpha }), \end{array}$$

2.5 $$\begin{array}{rl} \mu_{\beta ,\alpha } & ∼\text{Normal}(0,0.001), \end{array}$$

2.6 $$\begin{array}{rl} \varphi_{\beta ,\alpha } & =\frac{1}{\sigma_{\beta ,\alpha }^{2}}, \end{array}$$

2.7 $$\begin{array}{rl} \;\text{and}\;\sigma_{\beta ,\alpha } & ∼\text{Uniform}(0,10). \end{array}$$

Initially, we used simulated data to verify model structure. Due to uncertainty about where to set the initial parameter estimates, we ran the sampler multiple times to test for acceptable convergence. The final model was run using the entire dataset, initialized at the mean values from the previous run, and then run for 7500 iterations with a burn-in of 2000. Convergence and stationarity was assessed visually and by using Gelman and Geweke diagnostics [36]. We performed all analyses in Python, R v. 3.0.2, JAGS v. 3.4.0 and ArcGIS v. 10.2.

## 3. Results

We deployed 16 instrument packages on 15 monk seals with one adult male instrumented twice. We recovered data from all instruments (GPS, video and Open Tag) for six seals (five males and one female). We recovered Open Tag data without concurrent Crittercam and GPS footage for one animal (RA50). Due to issues with depth sensor calibration and/or malfunction, we included only six seals in the analysis (five with all data streams and one with Open Tag data only). Tracking duration ranged 2–5 days, covering at least two foraging trips for each seal. Summary statistics are presented as the mean ± s.d. The mean dive depth was 17.3 ± 16.8 m with search events occurring, on average, at 25.3 ± 16.2 m. An average of 482.8 ± 105.3 dives were recorded per seal, with 251.8 ± 169.3 search events detected per animal (table 2).

**Table 2.** Tracking duration and mean summary data for Hawaiian monk seals. Mean ± s.d. values for maximum dive depth, dive duration, dive ratio and search depth. Bottom time (%) is the percentage of dive time that the seal spent in the bottom portion of the dive. Search depth is the mean bathymetry during dives that contained search events. Dive ratio is the mean ratio of the maximum depth of the dive to the bathymetry where the dive occurred.

seal ID	island	no. dives	no. trips	no. days	dive depth (m)	dive duration (min)	bottom time (%)	dive ratio	search depth (m)
R012	Oahu	524	3	6	24.76 ± 13.96	7.0 ± 3.1	76.5	0.94 ± 0.18	27.5 ± 11.5
RA50	Oahu	502	n.a.	7	16.49 ± 15.71	4.3 ± 2.9	58.4	n.a.	n.a.
RV18	Kauai	575	8	6	7.6 ± 5.10	3.7 ± 2.6	55.2	0.62 ± 0.25	14.0 ± 5.1
RW02	Kauai	554	5	2	11.23 ± 9.03	3.8 ± 2.4	56.5	0.38 ± 0.29	35.7 ± 14.7
RM38	Molokai	457	17	5	25.24 ± 25.36	7.0 ± 3.3	66.1	0.65 ± 0.29	68.1 ± 34.7
R306	Molokai	372	4	3	32.93 ± 17.32	5.5 ± 1.5	72.0	0.80 ± 0.37	18.3 ± 15.9

### 3.1. Identifying search events

**Figure 3.**
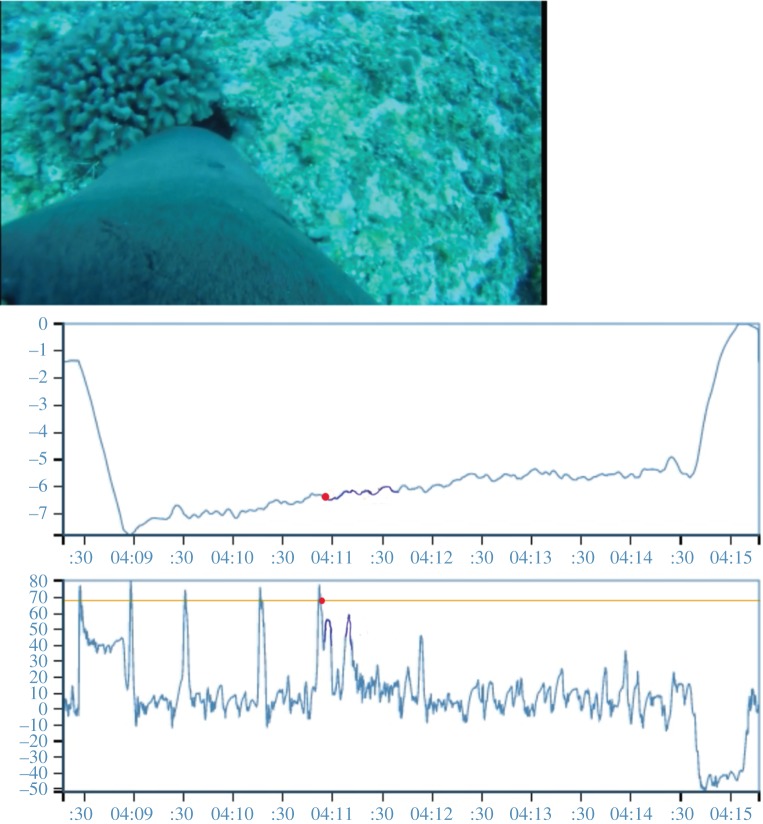
Screen shot of the animated pitch metric (electronic supplementary material, S1). This shows the movement axes from the Open Tag with concurrent video footage highlighting a peak in pitch as the animal searches for prey. The top graph shows Depth versus Time over the course of a dive and the bottom graph shows Pitch versus Time. All peaks in the pitch axis that occurred deeper than −3 m were recorded as search events. See electronic supplementary material, S1 for the full animation of this dive showing depth, pitch and concurrent video footage.

**Figure 4.**
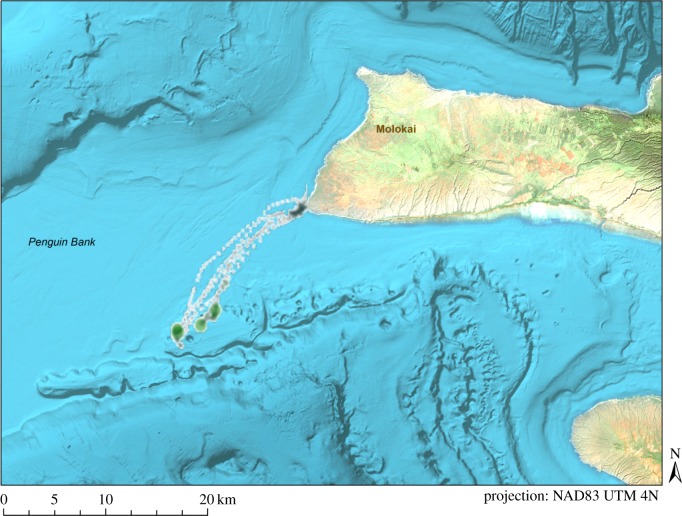
Foraging behaviour for RM38, a Hawaiian monk seal instrumented on Molokai. The size of the circles represents the amount of time spent in an inverted body position during a dive, i.e. the amount of time spent searching/foraging. There was some searching on all dives, but more time was spent foraging on the edge of Penguin Bank. The colour of the circles represents the distance from the shore, moving from white (near shore) to green (offshore).

We identified 3138 dives on the Open Tag for the six seals. Five of these seals had concurrent video footage, but the time stamps for one Crittercam malfunctioned and hence video matching was possible only for four seals. Ninety-three dives (3%) were matched to Crittercam footage to validate the pitch metric (figure 3; electronic supplementary material, S1). Two seals had one false-positive prediction each; the other two seals had a number of false negatives, but no false positives. Overall, the combination of pitch and depth was 78% successful at predicting dives that contained search events for monk seals (table 3). According to this metric, search events occurred on most dives, but some seals did spend more time foraging in particular areas. For example, figure 4 shows that although most dives for this animal contained search events (size of the circles), it spent more time actively foraging on dives that occurred on the edge of Penguin Bank.

**Table 3.** Predicted (via pitch) versus observed (via video) search events for monk seals in the MHI.

		predicted							
		R306		RM38		RW02		RV18	
		search	not	search	not	search	not	search	not
observed	search	2	8	0	9	12	0	8	0
	not	0	26	0	20	1	2	1	0

### 3.2. Model selection

The final model included the following predictors of search events: dive duration, mean ODBA, bathymetry, dive ratio, bottom % and distance to shore. Seal was included as a random effect. None of the highest posterior density intervals spanned zero, indicating that every predictor differed from zero in its influence on search events. Coefficient estimates (table 4) from the model output were used to predict the probability of a search event occurring on a dive. Generally, the probability of a search event increased as dive duration, depth and mean ODBA increased, and when seals performed benthic, square-shaped dives.

**Table 4.** Estimated coefficients and corresponding interpretation for the fixed effects of a mixed model looking at the influence of covariates on search events for Hawaiian monk seals in the MHI.

covariate	*β* estimate	s.e.	explanation
bathymetry	−0.038	0.007	probability of searching increases at deeper depths
shore distance	0.016	0.0053	probability of searching increases farther offshore
dive duration	0.66	0.041	longer dives increase the probability of searching
ODBA	9.077	1.08	more body motion during a dive increases the probability of searching
dive ratio	2.65	0.31	maximum depths closer to the sea floor increase the probability of searching
bottom %	1.15	0.28	increased time at the bottom of the dive increases the probability of searching

We expected that some measure of benthic complexity would predict search events, but model selection did not retain any of those variables (terrain ruggedness or rugosity). Habitat type was also excluded. Measures of bathymetry and dive ratio were included in the mixed model, but the other measures of bottom complexity were not. Light level (day or night) was not a good predictor of search events, even though 70% of all dives occurred during daylight.

## 4. Discussion

By combining high-resolution behaviour data with environmental variables, we defined a metric to identify search events and gained insight into the covariates that influence foraging in the MHI. This allowed us to develop a better description of Hawaiian monk seal foraging behaviour in this region. The probability of searching increased on long, deep dives with more time at the bottom and with increased body motion. Active search events seldom occurred near shore, in shallow water or during short dives in which the seal did not reach the bottom.

These and similar variables have been used to predict underwater behaviour in a number of pinniped species, including harbour seals (*Phoca vitulina*) and grey seals (*Halichoerus grypus*). However, they are most often used indirectly to aid in dive classification, which is then used to infer behaviour [18,34,37,38]. The collection of multiple high-resolution and simultaneous data streams allowed us to make more robust interpretations of underwater behaviour than two-dimensional classification allows.

### 4.1. Identifying foraging dives

As mentioned above, monk seals exhibited a single main dive type (square-shaped and benthic). With just one shape, it would be difficult to use statistical classification, such as kmeans, to separate dive types and infer function. Therefore, we used the pitch metric to identify search events, allowing us to define foraging dives without using traditional classification methods. We could not completely eliminate error associated with our prediction of search events, but this direct approach allowed us to validate a subset of the data and quantify the success of the approach. Other methods, including ODBA and jerk signals, use accelerometer data for dive classification and to infer dive function, but the pitch metric proved to be an accurate and straightforward method that revealed both the search and capture components of foraging.

#### 4.1.1. Overall dynamic body acceleration

ODBA is often used as a link to energy expenditure in animals fitted with accelerometers [20,39–41], but it is also a metric of an animal's overall body movement. Video footage revealed that the tagged monk seals increased their total body motion during prey-capture attempts. This was done to maintain the inverted body position and search for or attack prey. These events were also visible in just one axis due to the inverted body position (pitch, figure 4). ODBA is capable of measuring body motion in all dimensions, and hence when a seal attempted to capture or was handling prey, ODBA increased synergistically with total body motion. In an attempt to directly measure foraging effort, ODBA was initially included in our models as a link to energy expenditure. Our thought was that there would be an increase in ODBA on foraging dives, when compared to non-foraging dives. However, since some foraging dives (defined with the pitch metric) did not include capture attempts or searching that resulted in an increase in mean ODBA, we realized that this variable was not a good predictor of general foraging behaviour. For monk seals in this study, ODBA was a better indicator of total body motion and potential capture events than as a metric of general foraging behaviour, which includes both search and capture attempts.

#### 4.1.2. Jerk signals

Jerk signals [42,43] have also been used to identify prey capture events in pinnipeds and other marine mammals. A jerk signal is similar to ODBA in that it uses acceleration in all three axes, but also signifies the occurrence of rapid changes in motion (jerks). In monk seals, a jerk signal may miss the smooth transitions between swimming horizontally along the sea floor and a vertical position used to investigate/search for prey in specific locations. Additionally, the placement of the accelerometer and the sampling rate of the processor dictate the accuracy of this metric in identifying capture attempts. To identify head snaps or head shaking of a seal while foraging, the sampling rate must be high and the accelerometer is typically placed on the head or jaw. In harbour seals, Ydesen *et al*. [43] showed a 90% success rate in identifying both raptorial and suction-feeding using a 95 Hz sampling rate from a small, head-mounted accelerometer. We initially sampled at 100 Hz so that this type of analysis would be possible with monk seals, using back-mounted accelerometers. However, after watching the video footage, it became apparent that jerk signals could miss search events and we were interested in both search events and prey captures. Additionally, more work is needed to separate the signal of capture attempts from successes, which is beyond the scope of this paper. As a result, pitch was the most easily computed metric to classify foraging dives that included both search events and prey-capture attempts.

#### 4.1.3. Pitch metric

The pitch metric was imperfect and we identified false-negative search events for two seals. False-negative prediction results in underestimation of search behaviour and thus our analysis was conservative. With more than 75% prediction accuracy for this metric, we are confident that the effect of each predictor would remain the same even if all false negatives were removed. Additionally, we believe these false negatives resulted from instrument placement or an overly conservative definition of the metric. The pitch metric identified a search event when the pitch axis of the accelerometer peaked above 70°. Video footage showed an inverted body position for all search events, but if the seals were searching at a shallower angle, or if the tag was tilted after hitting a rock, the 70° peak may not have picked up all events. By adjusting the metric to include inversions at a shallower angle (i.e. 60°), most of the false negatives would probably be eliminated and we could eliminate this error. Additionally, with correct placement and known orientation of the Open Tag, we believe the number of false negatives would decrease and the accuracy of this metric would increase.

### 4.2. Environmental and behavioural influences on foraging

Documenting where and when foraging events occur is an important step to understanding monk seal foraging behaviour in the MHI. Additionally, these data will assist management and recovery efforts by allowing the public and other concerned stakeholders who may view monk seals as competitors to see for themselves where, when and how monk seals are foraging throughout the MHI. Wilson [25] suggested that monk seals were actively searching for prey the entire time they were at sea. These results support that hypothesis and expand on it to describe the variables that influence monk seal foraging behaviour in the MHI.

#### 4.2.1. Depth

Dive depth and bathymetry were both good predictors of search events, with the probability of a search decreasing in shallower water. Monk seals swim near the bottom almost exclusively while at sea [25,32,44]; consequently, measures of dive depth and bathymetry were similar, potentially making these two depth variables functionally redundant. In the final model, dive depth was excluded, but bathymetry and dive ratio (the ratio of dive depth to bathymetry) were retained. By including both dive ratio and bathymetry (correlation: 0.088), which tells us the available depth and how close the seals were to that depth, we essentially used a similar metric to dive depth, but accounted for more variation in the data and gained additional information regarding preferred foraging depths.

#### 4.2.2. Duration

Dive duration is commonly used to classify and analyse foraging behaviour in many diving predators [6,8,38,45,46]. Unsurprisingly, as dives became longer in our study, the probability of a search event increased. Monk seals swam in a near-vertical orientation as they descended to the sea floor, with little horizontal movement in the descent and ascent phases of the dive. Therefore, monk seals spent most of their submerged time travelling along the sea floor where they might encounter prey. Consequently, an increase in dive duration resulted in an increase in bottom time. The proportion of time that a seal spends at the ‘bottom’ of a dive (bottom %) has long been considered a metric of ‘foraging effort’ [38]. This variable measures the amount of time (effort) a seal expends in the search for potential prey. Longer dives and more time spent at the bottom of a dive would expose seals to more potential prey. Consequently, the probability of searching increased on longer dives with more time spent at the bottom.

#### 4.2.3. Body motion

As mentioned previously, ODBA is often used as a link to energy expenditure and foraging effort over the course of a dive; however, in this study, it was a better indicator of total body motion. We used the mean ODBA over the course of a dive as a predictor of foraging behaviour, assuming that increased body motion was related to active foraging behaviour. When a seal stopped to search for potential prey (inverted position) but did not find a prey item, the seal quickly continued along its path. However, if the seal attacked a potential prey item or expended more effort into searching at that location, it employed greater movements of the flippers and made changes in orientation to maintain a body position capable of removing the prey from its hiding place, thus increasing total body motion. ODBA reflects movement in all three dimensions, an increase in which would only occur in the latter scenario, if a seal was actively pursuing a prey item. Therefore, an increase in the mean ODBA on a dive resulted in an increased probability of a search event on that dive. This result suggests that in addition to being a good predictor of search events for any seal, in the future we may also be able to use ODBA, or some other measure of overall body motion, to separate searching from prey-capture attempts. However, it will be important to identify a signal that separates a thorough search event (digging/rock flipping) from an actual capture attempt.

#### 4.2.4. Time of Day

Most dives (70%) occurred during daylight hours so we expected to see some diel pattern for foraging. However, light was not a good predictor of search events for any seal, suggesting that there are a number of reasons why a seal may leave the beach, including disturbance or thermoregulation, which are not directly related to foraging behaviour.

#### 4.2.5. Habitat

We observed seals digging in the sand or turning over rocks and coral heads to find cryptic prey, so terrain ruggedness (complexity) or a sandy substrate are necessary for their foraging strategy to be successful. Habitats visited by the seals in this study were often defined as ‘pavement’ or ‘aggregate reef’. ‘Pavement is flat, low-relief, solid carbonate rock covered by macroalgae, hard coral, zoanthids and other sessile invertebrates that are dense enough to begin to obscure the underlying surface’ [31]. This definition describes a substrate that is likely to be too dense for prey to burrow into, and lacks relief such as large coral heads. ‘Aggregate Reef is an area of high relief that lacks sand channels of spur and groove’ [31]. This habitat would provide more hiding places for potential prey, but the complexity of the habitat may require too much effort for the location and capture of prey to make it worthwhile. Neither habitat seems ideal for monk seal foraging. Consequently, habitat structure was not a significant predictor in our model, either because there was not enough variation in habitat between foraging and non-foraging dives, or perhaps because habitat classification in this region needs more refinement before it can be useful on a fine spatial scale. On the level of individual dives, seals may encounter coral heads or small caves that are otherwise surrounded by ‘pavement’. The 1 km resolution of the habitat grid could miss small areas of relief, making it difficult to use this habitat classification at a fine spatial scale. Additionally, the occurrence of boulders, coral heads and other relief may be one of the drivers behind the seals' strategy of foraging continuously while at sea. These areas are not clustered together in ‘hot spots’, which forces the seals to continuously and opportunistically search for prey while at sea instead of travelling directly to specific areas every time they enter the water.

## 5. Conclusion and future directions

Hawaiian monk seals in the MHI searched for prey on longer dives in which they exhibited an increase in body motion. Seals searched for prey primarily in deeper waters farther offshore and the probability of a search event could be predicted by some combination of depth, duration and body motion. Wilson [25] and Cahoon [24] both showed that monk seals in the MHI have shorter foraging trips than their counterparts in the NWHI. Combining these previous results with those reported here supports the hypothesis that monk seals in the MHI are able to forage more efficiently, or with less effort, than those in the NWHI. Monk seals in the MHI have shorter foraging trips, but most of their dives contain search events, suggesting that these seals are continually searching for prey while at sea, and that they are able to find and acquire the necessary resources with less effort/time than those in the NWHI. The ecological implications of these results are still unclear. Previous studies have shown that the diets between the two regions are similar [33,34], but the resolution (family-level) of the prey identification in these studies is not fine enough to detect differences in the effort required to capture the prey (e.g. the effort required to target deep-water bottom fish versus reef bottom fish). In addition, we still do not know whether prey quality is better so that seals can acquire sufficient resources through fewer prey items, if prey are more abundant because competitors have been removed by human fishing efforts or if the removal of the competitors themselves has eliminated direct interspecific competition in the MHI. Comparison of the video footage between the two regions suggests that there is significantly less competition for seals in the MHI than in the NWHI. Monk seals in the NWHI were ‘escorted’, on average, by roughly 17 competitors (such as sharks and large jacks) while foraging [44], but in the MHI only two of the tagged seals were observed with a small number (1–2) of escorts. With less competition, seals may be able to take advantage of readily accessible habitat in the MHI, resulting in greater time spent foraging and less total time spent at sea.

We can now identify where monk seals are foraging in the MHI and what covariates structure foraging behaviour in this region. We hope to use this knowledge to inform management strategies to aid in outreach and recovery efforts. The results of this study provide the second step (a metric to identify foraging events) in our study of Hawaiian monk seal behaviour and what it takes, energetically, to succeed as a monk seal in Hawaii. The next steps will be to use this information to determine how foraging behaviour varies among age and sex classes in the MHI, and to document more prey-capture events with video cameras and accelerometers so that we can develop metrics to separate search and capture events.

Accelerometers are becoming smaller and smaller as technology improves, but it is still not possible to transmit accelerometer data via satellite or GSM networks. However, the pitch metric described here is relatively simple to calculate with inversions easily identified using peaks in the data. Theoretically, this calculation could be included in the on-board processing of dive summaries and transmitted via satellite or GSM networks. This type of on-board processing would enable researchers studying benthic-foraging pinnipeds to separate foraging from non-foraging dives without having to recover the accelerometers.

## Data accessibility

The data accompanying this manuscript will be made available through the Dryad digital repository: http://dx.doi.org/10.5061/dryad.s0b80 [47].
